# Look who is calling: a comparison of genotype calling algorithms

**DOI:** 10.1186/1753-6561-3-s7-s59

**Published:** 2009-12-15

**Authors:** Maren Vens, Arne Schillert, Inke R König, Andreas Ziegler

**Affiliations:** 1 Institut für Medizinische Biometrie und Statistik, Universität zu Lübeck, 23538 Lübeck, Germany

## Abstract

In genome-wide association studies, high-level statistical analyses rely on the validity of the called genotypes, and different genotype calling algorithms (GCAs) have been proposed. We compared the GCAs Bayesian robust linear modeling using Mahalanobis distance (BRLMM), Chiamo++, and JAPL using the autosomal single-nucleotide polymorphisms (SNPs) from the 500 k Affymetrix Array Set data of the Framingham Heart Study as provided for the Genetic Analysis Workshop 16, Problem 2, and prepared standard quality control (sQC) for each algorithm. Using JAPL, most individuals were retained for the analysis. The lowest number of SNPs that successfully passed sQC was observed for BRLMM and the highest for Chiamo++. All three GCAs fulfilled all sQC criteria for 79% of the SNPs but at least one GCA failed for 18% of the SNPs. Previously undetected errors in strand coding were identified by comparing genotype concordances between GCAs. Concordance dropped with the number of GCAs failing sQC. We conclude that JAPL and Chiamo++ are the GCAs of choice if the aim is to keep as many subjects and SNPs as possible, respectively.

## Background

A crucial step in the data generation process of genome-wide association studies is genotype calling. Here, qualitative genotypes are derived from measured signal intensities of the two alleles of a single-nucleotide polymorphism (SNP). Because missing or erroneous genotypes can flaw the high-level statistical association analysis, a series of different genotype-calling algorithms (GCAs) have been proposed [1].

The outcome of these GCAs can differ substantially [2]. We therefore compared different GCAs using the genotype data from participants of the Framingham Heart Study SNP Health Association Resource project. We investigated the influence of GCAs on autosomal SNPs that passed the filtering by errors in strand coding and standard quality control (sQC).

## Methods

Hybridization probe intensity CEL data of 6,848 participants in the Framingham Heart Study was provided as Problem 2 for the Genetic Analysis Workshop 16 (GAW16) [3]. Genotyping was performed using the Affymetrix GeneChip^® ^Human Mapping 500 k Array Set.

We limited our analyses to the 2,466 participants of the 332 families with complete genotypes in the nuclear families.

Three different GCAs were considered for comparison. Bayesian robust linear modeling using Mahalanobis distance (BRLMM) has been recommended by the manufacturer for the 500 k Array Set [4]. Chiamo++ (Italian for "I call") uses a Bayesian hierarchical four-class mixture model [5]. JAPL (French for "I call") is based on an expectation-maximization (EM) clustering algorithm that was improved by Plagnol et al. [6]. Where probe intensities had to be normalized beforehand, CelQuantileNorm was used [7]. Normalization had to be split in two parts because of memory access errors when more than approximately 2,000 samples were used in one run. The data were split randomly in two batches of similar size. Chiamo++ and JAPL were run using default settings, BRLMM calls were used as provided for GAW16.

Only those SNPs provided in the GAW16 BRLMM data set were used for further analysis. Furthermore, X-chromosomal SNPs and SNPs with different strand codings in the GCAs were excluded.

**Figure 1:**
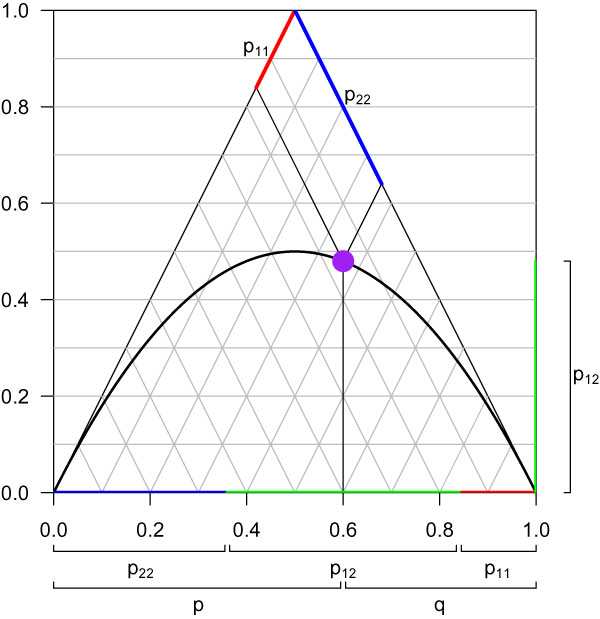
**The deFinetti triangle**. The deFinetti triangle shows genotype frequencies and allele frequencies for each SNP. For example, the purple point displays one SNP. The two homozygote genotype frequencies for a SNP are read as length of the projections along the sides of the triangle (shown in red and blue). The proportion of heterozygotes (green) is given either on the vertical axis or as difference between 1 and the homozygous frequencies. The allele frequencies are shown on the horizontal axis. The curve displays the genotype distributions that are exactly in HWE.

For the remaining SNPs, deFinetti triangles presenting allele and genotype distributions were generated for all three GCAs (an example for a deFinetti triangle is given in Figure 1). In these, the two homozygote genotype frequencies for each SNP are read as length of the projections along the sides of the triangle, and the allele frequencies and the proportion of heterozygotes are given on the horizontal and vertical axes [8].

Samples with a call fraction <97% were excluded, and sQC was performed separately for all three GCAs. Specifically, SNPs were excluded if the exact lack-of-fit test for Hardy-Weinberg equilibrium (HWE) revealed *p *< 10^-4^, if the minor allele frequency (MAF) was <1%, or if the missing frequency (MiF) was <2%.

**Figure 2:**
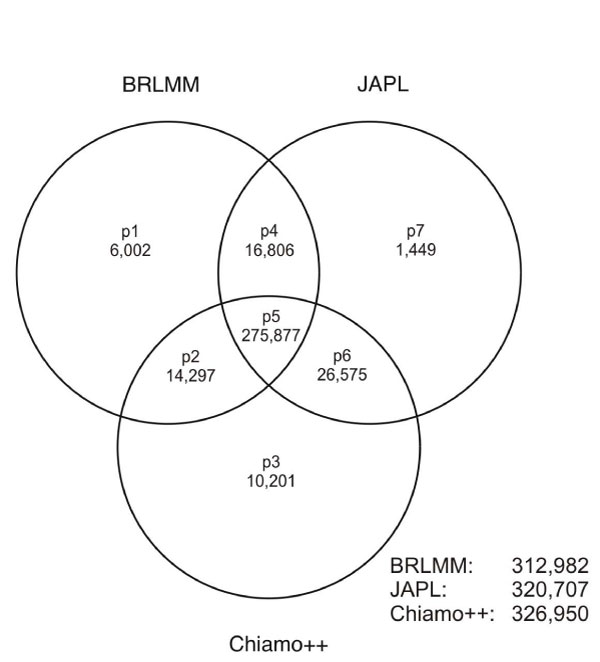
**Venn diagram of the SNPs passing sQC after different calling algorithms**. SNPs in different groups p1 to p7 passed sQC for different combinations of calling algorithms, e.g., SNPs from group p1 only passed sQC for BRLMM, whereas in group p5 SNPs passed sQC for all three algorithms. In addition, the Venn diagram gives the number of SNPs passing sQC in each calling algorithm. A total of 351,207 SNPs out of 422,655 SNPs passed the sQC for at least one calling algorithm.

We defined seven different groups of SNPs after sQC according to Figure 2 and investigated the characteristics of SNPs in each group. A detailed analysis of all SNPs was computationally impossible because this would have required a comparison of >3 billion genotypes. We therefore drew a random sample of 10,000 SNPs from group p5 and a random sample of 1,000 SNPs for every other group.

We termed an individual to be concordant for the considered GCAs if the GCAs yielded the same result (genotype or missing) for the specific SNP. We then derived concordance fractions on the SNP level. Confidence intervals were estimated as 95% exact Blyth-Still-Casella confidence intervals (95% CI).

Analyses were performed in the statistical package R, version 2.7.1, with the GenABEL, version 1.4-1 library [9]. The analyses were carried out on an Intel Quad-Core Dual Xeon E5345 computer with a 2.33 GHz processor, 32 GB RAM, and a 64-bit SUSE Enterprise Linux operating system.

## Results and discussion

**Figure 3:**
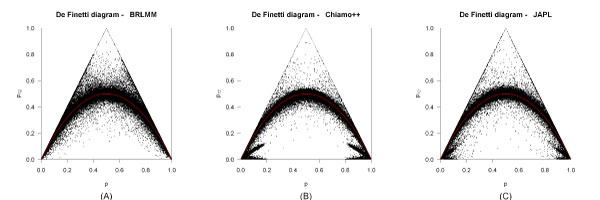
**DeFinetti triangles for the genotype calling algorithms BRLMM, Chiamo++, and JAPL**. Genotype distributions of all 422,655 SNPs that fulfilled the filtering criteria prior to standard quality control (sQC). A, the genotype distributions for BRLMM; B, genotype distributions for Chiamo++; C, genotype distributions for JAPL.

A total of 486,605 SNPs were provided for GAW16. We excluded 63,950 SNPs because of different strand coding or allele flips, leaving 422,655 SNPs for further analysis. The deFinetti triangles (Figure 3) give an overview of the genotype distribution of these SNPs. BRLMM and JAPL showed excess heterozygosity, i.e., more heterozygous subjects than expected under HWE, for a larger number of SNPs than Chiamo++ (BRLMM, 56.04%; Chiamo++, 53.66%; JAPL, 56.74%). Both algorithms revealed many SNPs with a high number of heterozygous subjects but low frequency for one of the homozygous genotypes.

In contrast, Chiamo++ more often led to genotype distributions at the boundary of the deFinetti triangle than BRLMM and JAPL (BRLMM, 19.77%; Chiamo++, 23.83%; JAPL, 20.06%), where the boundary was defined by a frequency of <2 × 10^-3 ^for one genotype group per SNP without counting monomorphic SNPs. Interestingly, Chiamo++ yielded SNPs with an extremely low heterozygosity (<2 × 10^-3^) more often than BRLMM and JAPL (BRLMM, 1.84%; Chiamo++, 5.39%; JAPL, 1.95%). These SNPs fell in one of two groups: The first had a MAF lower than 20% but only 2.5% heterozygous subjects. For the second, one could imagine that they form the rudiment of a second curve with maximum at the point (0.5; 0.25). As in the first group of SNPs, this curve was only observed for SNPs with MAF<20%. Because this curve is usually seen for X-linked SNPs if males and females are pooled, we investigated the genotype frequencies for a series of these SNPs by sex but we detected no differences.

The call fraction was >0.97 for all individuals in JAPL. Five subjects were excluded using Chiamo++. BRLMM called these five participants and an additional four with a fraction of <0.97. This finding is in line with the conclusions drawn by the developers of JAPL, who state that their algorithm was specifically designed to deal with uncertain genotypes which are said to be missing by other GCAs [6].

Based on the subset of individuals who passed sQC for all three GCAs, the observations from the deFinetti triangles are confirmed by results of sQC (Table 1): for Chiamo++, twice as many SNPs failed the HWE criterion. Most SNPs failed due to MAF criterion in Chiamo++, but rates are comparable among all three algorithms. For BRLMM and JAPL, most SNPs failed the MiF criterion. Interestingly, BRLMM removed almost twice as many SNPs as Chiamo++ through this criterion.

**Table 1:** Overview of sQC

	No. SNPs removed (%)		
	
QC criteria	BRLMM	Chiamo++	JAPL
Failed MAF	37,259 (8.85%)	44,181 (10.45%)	39,201 (9.27%)
Failed MiF	76,528 (18.11%)	41,939 (9.92%)	62,969 (14.90%)
Failed HWE	7,519 (1.78%)	16,891 (4.00%)	7,208 (1.71%)
Passed all sQC	312,982 (74.05%)	326,950 (77.36%)	320,707 (75.88%)

In total, the highest number of SNPs fulfilling all sQC criteria was obtained using Chiamo++ (77.36%) and the smallest number was obtained using BRLMM (74.05%). 351,207 SNPs (83.10%) passed the sQC in at least one algorithm. Of these SNPs, 78.55% fulfilled all sQC criteria for all three GCAs jointly (Figure 2).

In summary, if the aim is to keep as many subjects as possible for analysis, which is of interest in genome-wide association studies with a small sample size or in family-based genome-wide association studies, JAPL would be the GCA of choice. Chiamo++ would be preferred if one aims at keeping a high number of SNPs for further analysis.

Results of the concordance estimation are summarized in Table 2. In group p5, six SNPs showed a concordance <0.52. All other SNPs had a concordance >0.89. The six SNPs with low concordance had a MAF~50%, and all were either GC or AT SNPs, so these represented errors in strand codings that were not detected in the filtering step. Because these SNPs were identified in a random sample of 10,000 SNPs, the total number of SNPs that fulfilled all sQC criteria but had undetected errors in strand coding is expected to be 165.53 (95% CI: 82.76-358.64).

**Table 2:** Concordance of calling algorithms

SNPs from group^a^	Concordance between	Minimum concordance
p1	BRLMM-JAPL	0.3215
p1	BRLMM-Chiamo++, without allele flips	0.7725
p1	BRLMM-Chiamo++	0.486
p1	BRLMM-Chiamo++-JAPL	0.3158
p2	BRLMM-Chiamo++, without allele flips	0.9202
p2	BRLMM-Chiamo++	0.466
p2	BRLMM-Chiamo++-JAPL	0.3663
p3	Chiamo++-BRLMM	0.4933
p3	Chiamo++-JAPL	0.1848
p3	Chiamo++-JAPL-BRLMM	0.1787
p4	BRLMM-JAPL	0.9683
p4	BRLMM-JAPL-Chiamo++	0.781
p5	BRLMM-Chiamo++-JAPL, without allele flips	0.8987
p6	JAPL-Chiamo++	0.4648
p6	JAPL-Chiamo++-BRLMM	0.4628
p7	JAPL-BRLMM	0.3952
p7	JAPL-Chiamo++	0.7037
p7	JAPL-BRLMM-Chiamo++	0.4888

^a^p1 to p7 are used according to Figure 2.

There were two SNPs in group p2 that had a concordance <0.48. Both SNPs had a MAF~50% and were GC SNPs. All other SNPs in this group had a concordance >0.92. In p4, all SNPs had a concordance >0.96. In p6, the concordance was only >0.46, but we were not able to detect the cause.

In general, estimating concordance with one or more GCAs failing sQC led to considerably lower values. Specifically, we found dramatically low concordance fractions (minimum concordance fractions between 18% and 78%) for SNPs that did not pass sQC in all considered GCAs. This might be due to the fact of disagreement in calling genotypes as "missing".

## Conclusion

Among the investigated GCAs, JAPL is recommended if the aim is to keep as many subjects as possible for analysis. Chiamo++ would be preferred if the number of SNPs for further analysis needs to be high. By comparing the concordances between different calling algorithms, otherwise-undetected errors in strand coding were identified. Considering SNPs that did not pass the sQC in at least one of the considered algorithms, the concordance frequency is considerably lower.

## List of abbreviations used

BRLMM: Bayesian robust linear modeling using Mahalanobis distance; EM: Expectation maximization; GAW16: Genetic Analysis Workshop 16; GCA: Genotype-calling algorithms; HWE: Hardy-Weinberg equilibrium; MAF: Minor allele frequency; MiF: Missing frequency; SNP: Single-nucleotide polymorphism; sQC: Standard quality control.

## Competing interests

The authors declare that they have no competing interests.

## Authors' contributions

MV participated in the design of the study, did the calling, prepared the figures, and drafted the manuscript. AS participated in the design of the study and figures, and did the sQC. IRK participated in the design of the study and its coordination and helped to draft the manuscript. AZ conceived of the study, participated in its design, and helped to draft the manuscript. All authors read and approved the final manuscript.

## References

[B1] Teo YY Common statistical issues in genome-wide association studies: a review on power, data quality control, genotype calling and population structure Curr Opin Lipidol 2008 19 133 143 10.1097/MOL.0b013e3282f5dd77 18388693

[B2] Samani NJ Erdmann J Hall AS Hengstenberg C Mangino M Mayer B Dixon RJ Meitinger T Braund P Wichmann HE Barrett JH König IR Stevens SE Szymczak S Tregouet DA Iles MM Pahlke F Pollard H Lieb W Cambien F Fischer M Ouwehand W Blankenberg S Balmforth AJ Baessler A Ball SG Strom TM Braenne I Gieger C Deloukas P Tobin MD Ziegler A Thompson JR Schunkert H for the WTCCC and the Cardiogenics Consortium Genome-wide association analysis of coronary artery disease N Engl J Med 2007 357 443 453 17634449 10.1056/NEJMoa072366 PMC2719290

[B3] Cupples LA Heard-Costa N Lee M Atwood LD Genetic Analysis Workshop 16 Problem 2: The Framingham Heart Study Data BMC Proc 2009 3 suppl 7 S3 10.1186/1753-6561-3-s7-s3 PMC2795927 20018020

[B4] Affymetrix BRLMM: An improved genotype calling method for the GeneChip^® ^Mapping 500K Array Set http://affymetrix.com/support/technical/whitepapers/brlmm_whitepaper.pdf

[B5] Wellcome Trust Case Control Consortium Genome-wide association study of 14,000 cases of seven common diseases and 3,000 shared controls Nature 2007 447 661 678 17554300 10.1038/nature05911 PMC2719288

[B6] Plagnol V Cooper JD Todd JA Clayton DG A method to address differential bias in genotyping in large-scale association studies PLoS Genet 2007 3 e74 17511519 10.1371/journal.pgen.0030074 PMC1868951

[B7] CelQuantileNorm http://www.wtccc.org.uk/info/software.shtml

[B8] Ziegler A König IR A Statistical Approach to Genetic Epidemiology: Concepts and Applications 2006 Weinheim, Wiley-VCH

[B9] Aulchenko YS Ripke S Isaacs A van Duijn CM GenABEL: an R library for genome-wide association analysis Bioinformatics 2007 23 1294 1296 10.1093/bioinformatics/btm108 17384015

